# Neglected disease, neglected populations: the fight against *Cryptococcus* and cryptococcosis

**DOI:** 10.1590/0074-02760180111

**Published:** 2018-04-12

**Authors:** Marcio L Rodrigues

**Affiliations:** Instituto Carlos Chagas Fundação Oswaldo Cruz (Fiocruz)

Recent estimates indicate that the diseases caused by the fungal pathogens *Cryptococcus neoformans* and *C. gattii* kill more than 180,000 humans per year (Rajasingham et al. 2017), which ranks cryptococcosis in absolute numbers of deaths as the 5th most lethal infectious disease, behind AIDS, tuberculosis, malaria and diarrhea (WHO 2016, WHO 2017, WHO 2018). *Cryptococcus* infects humans through inhalation and, mostly in immunosuppressed patients, the fungus reaches the brain to cause a highly lethal meningitis (Colombo and Rodrigues 2015). The most efficient anti-cryptococcal therapy, developed in the 1950s, is based on the use of the polyene antifungal amphotericin B, which is toxic intravenous (Williamson 2017). Amphotericin B is not available in most of the African continent (GAFFI - Available from: https://http://www.gaffi.org), where most of the individuals suffering from cryptococcosis are concentrated (Rajasingham et al. 2017). In Africa, antifungal resistance to azoles has reached alarming numbers (Mpoza et al. 2017). As recently pointed out (Williamson 2017), cryptococcal meningitis rates have shown no reductions over 2010-2014 in Botswana, despite nearly achieving 90% HIV detection, 90% enrolment in therapy, and 90% achievement of undetectable viral loads (Tenforde et al. 2017). Antifungal vaccines approved for human use are still not available.

Innovative tools to prevent or fight cryptococcosis are clearly necessary, but the disease is currently immersed in worrying circumstances. Cryptococcosis ranks amongst the most poorly funded neglected diseases in the world, receiving less than 0.5% of available relevant research and development funding (Rodrigues 2016). The disease is not even recognised by the World Health Organization as a neglected tropical disease (Molloy et al. 2017), which undermines the establishment of governmental and institutional programs of research funding. Research and development on *Cryptococcus* and cryptococcosis is still excessively focused on basic research (Albuquerque and Rodrigues 2012, Colombo and Rodrigues 2015), which might be related with the funding adversities faced by the field.

Despite all difficulties and still reduced size, the cryptococcal community has been continuously producing high-level scientific knowledge (Kwon-Chung et al. 2012). Well-known for having constructed a friendly community, scientists in the field have been actively discussing advances in the area since 1989, when Jerusalem (Israel) held the 1st Conference on *Cryptococcus* and Cryptococcosis (ICCC). Subsequent meetings were held in Milan (Italy, 1993), Paris (France, 1996), London (United Kingdom, 1999), Adelaide (Australia, 2002), Boston (USA, 2005), Nagasaki (Japan, 2008), Charleston (USA, 2011) and Amsterdam (Netherlands, 2014). In its 10th edition (2017), the ICCC has come to the developing world for the first time, being held in Foz do Iguaçu (Brazil, 2017). Besides the unquestionable scientific advances discussed during the meeting in Brazil, which are detailed in this special issue, the outcome of the 10th ICCC could not be more exciting: the 2020 conference will be held for the first time in Africa, in Uganda.

The scenario behind *Cryptococcus* and cryptococcosis is unquestionably complex, but the cryptococcal community has been actively expanding its potential to generate advances in science and scientific policies. Cryptococcosis has been recently proposed as a tropical neglected disease (Molloy et al. 2017) and similarly positive initiatives need to be highlighted, as the early formation of the French Cryptococcosis Study Group, the establishment of the Cryptococcal Meningitis Action Group (CryptoMAG), and the formation of the Brazilian and Latin American networks of Cryptococcosis, among others. However, concrete actions from decision makers and public health authorities are urgent to revert the current situation of reduced funding and lack of innovation. The prolific debates that have been conducted by a mature and friendly scientific community over the last decades bring optimism to the field and the belief that soon novel solutions to combat and prevent cryptococcosis will be available.
